# Infectious Clones of Tomato Chlorosis Virus: Toward Increasing Efficiency by Introducing the Hepatitis Delta Virus Ribozyme

**DOI:** 10.3389/fmicb.2021.693457

**Published:** 2021-07-26

**Authors:** Elisa Navas-Hermosilla, Elvira Fiallo-Olivé, Jesús Navas-Castillo

**Affiliations:** Instituto de Hortofruticultura Subtropical y Mediterránea “La Mayora,” Consejo Superior de Investigaciones Científicas, Universidad de Málaga (IHSM-CSIC-UMA), Algarrobo-Costa, Málaga, Spain

**Keywords:** tomato chlorosis virus, *Closteroviridae*, criniviruses, infectious clones, hepatitis delta virus ribozyme, *Nicotiana benthamiana*

## Abstract

Tomato chlorosis virus (ToCV) is an emergent plant pathogen that causes a yellow leaf disorder in tomato and other solanaceous crops. ToCV is a positive-sense, single stranded (ss)RNA bipartite virus with long and flexuous virions belonging to the genus *Crininivirus* (family *Closteroviridae*). ToCV is phloem-limited, transmissible by whiteflies, and causes symptoms of interveinal chlorosis, bronzing, and necrosis in the lower leaves of tomato accompanied by a decline in vigor and reduction in fruit yield. The availability of infectious virus clones is a valuable tool for reverse genetic studies that has been long been hampered in the case of closterovirids due to their genome size and complexity. Here, attempts were made to improve the infectivity of the available agroinfectious cDNA ToCV clones (isolate AT80/99-IC from Spain) by adding the hepatitis delta virus (HDV) ribozyme fused to the 3′ end of both genome components, RNA1 and RNA2. The inclusion of the ribozyme generated a viral progeny with RNA1 3′ ends more similar to that present in the clone used for agroinoculation. Nevertheless, the obtained clones were not able to infect tomato plants by direct agroinoculation, like the original clones. However, the infectivity of the clones carrying the HDV ribozyme in *Nicotiana benthamiana* plants increased, on average, by two-fold compared with the previously available clones.

## Introduction

The family *Closteroviridae* includes almost 60 plant virus species grouped in the genera *Ampelovirus*, *Closterovirus*, *Crinivirus*, and *Velarivirus*, plus a number of unassigned species (Fuchs et al., 2020). Closterovirids have several distinguishing characteristics: (i) semi-persistent transmission by insects, (ii) mostly phloem-limited, (iii) elongated particles built of several proteins, (iv) large single-stranded positive-sense RNA (ssRNA) genomes of 15–20 kb, (v) presence of a gene homologous to the HSP70 family of cell chaperones, and (vi) a number of duplicated and diverged genes (Agranovsky, 2016). Some closterovirids cause serious diseases in important crops including citrus, cucurbits, grapevine, sugar beet, and tomato.

Criniviruses have segmented genomes encapsidated in separate long flexuous virions that are restricted to the phloem and companion cells (Kiss et al., 2013). With the sole exception of a tripartite crinivirus, potato yellow vein virus (Livieratos et al., 2004), all members of the genus have a bipartite genome composed of RNA1 and RNA2. Criniviruses are transmitted in nature by whiteflies (Hemiptera: Aleyrodidae) in a semi-persistent manner (Tzanetakis et al., 2013).

**FIGURE 1:**
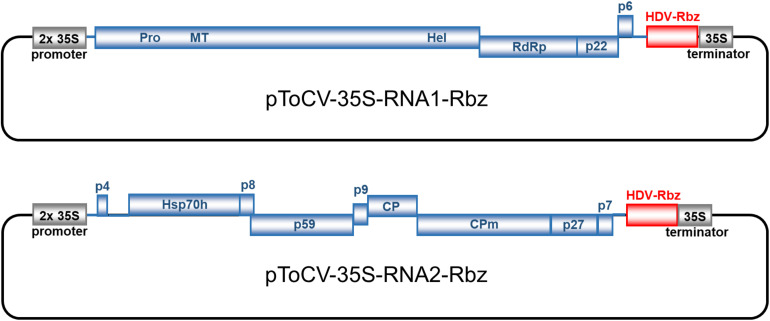
Genome organization and construction of infectious full-length cDNA clones of tomato chlorosis virus (Spanish isolate AT80/99-IC; RNA1, pToCV-35S-RNA1-Rbz; RNA2, pToCV-35S-RNA2-Rbz) in the binary vector pJL89 under the control of duplicated 35S cauliflower mosaic virus promoter (2 × 35S promoter) and incorporation of hepatitis delta virus ribozyme (HDV-Rbz). Blue boxes represent open reading frames with the putative protein products or protein domains indicated inside or above. PRO, protease; MT, methyltransferase; HEL, helicase; RdRp, RNA-dependent RNA polymerase; Hsp70 h, heat shock protein 70 homolog; CP, coat protein; CPm, minor coat protein.

Tomato chlorosis virus (ToCV) is a crinivirus first detected in Florida, United States, where it has caused a problem in greenhouse-grown tomatoes since 1989, the so-called “yellow leaf disorder” (Wisler et al., 1998b). Symptoms caused by ToCV include interveinal yellow chlorotic areas that initially develop on lower leaves and progress toward the upper leaves of the plant. Bronzing and red patches often occur within the yellow areas, and the leaves become thickened and crispy with the margins slightly curled upward. Fruit ripening is affected, resulting in economic damage. In addition to tomato, ToCV has a wide range of hosts, both natural and experimental, including some important crops such as pepper and potato (Fiallo-Olivé and Navas-Castillo, 2019). ToCV is transmitted in a semi-persistent manner by whiteflies of the *Bemisia tabaci* cryptic species complex (at least MEAM1, MED, and NW, formerly biotypes B, Q, and A, respectively), *Trialeurodes vaporariorum*, and *T. abutiloneus* (Wisler et al., 1998a; Navas-Castillo et al., 2000). Since first being detected in Florida, the presence of ToCV has been confirmed in 38 countries or territories of the Americas, Europe, Asia, and Africa (Fiallo-Olivé and Navas-Castillo, 2019; Amer et al., 2020; Kimathi et al., 2020; Raza et al., 2020), constituting a paradigmatic example of an emergent plant pathogen. The ToCV genome is composed of RNA1 (8,593–8,596 nt)-encoding proteins associated with virus replication and RNA2 (8,242–8,247 nt)-encoding proteins associated with encapsidation, cell-to-cell movement, and whitefly transmission (Wintermantel et al., 2005; Lozano et al., 2006, 2007; Figure 1). Both RNA segments contain gene-encoding proteins with RNA silencing suppression activity, the p22 protein in RNA1 and the major (CP) and minor (CPm) coat proteins in RNA2 (Cañizares et al., 2008).

Infectious plant virus clones are a useful tool for confirming Koch’s postulates, performing genetic reverse studies, and selecting plants in breeding programs. Viral DNA (or cDNA for RNA viruses) cloned within bacterial *Escherichia coli* plasmids can be used for *in vitro* transcription of viral RNA for the inoculation of plants (for RNA viruses) or for *in vivo* transcription after transformation into *Agrobacterium tumefaciens* and inoculation of plants by agroinfiltration (for RNA and DNA viruses). *In vivo* transcription from agroinfectious clones is usually under the control of the (duplicated) cauliflower mosaic virus (CaMV) 35S promoter and the *A. tumefaciens* nopaline synthase (NOS) terminator signal.

Currently, agroinfectious clones are available for eight closterovirids, the closteroviruses beet yellows virus (BYV) (Prokhnevsky et al., 2002), citrus tristeza virus (CTV) (Gowda et al., 2005; Ambrós et al., 2011, 2013), and grapevine leafroll-associated virus 2 (GLRaV-2) (Liu et al., 2009; Kurth et al., 2012), the criniviruses lettuce infectious yellows virus (LIYV) (Wang et al., 2009), lettuce chlorosis virus (LCV) (Chen et al., 2012), ToCV (Orílio et al., 2014; Zhao et al., 2016), and cucurbit chlorotic yellows virus (CCYV) (Shi et al., 2016), and the ampelovirus grapevine leafroll-associated virus 3 (GLRaV-3) (Jarugula et al., 2018).

Most of the agroinfectious closterovirid clones are not able to directly infect the natural hosts but require prior infection of *Nicotiana benthamiana* plants or protoplasts as an intermediate host. Then, the infectious (purified) virions can be transferred to the natural hosts to initiate a successful infection by different methods like bark-flap or stem slash inoculation (CTV; Gowda et al., 2005; Ambrós et al., 2011), whitefly transmission by membrane feeding (LIYV; Wang et al., 2009), or grafting (ToCV; Orílio et al., 2014). An exception is the case of an agroinfectious clone of GLRaV-2 that is able to directly infect young micropropagated grapevine plants (Kurth et al., 2012).

In most cases examined, non-viral nucleotides at the 5′ extremity of *in vivo* transcripts generated from infectious plant RNA virus clones greatly diminished infectivity (Boyer and Haenni, 1994). Although not as critical as the 5′ ends, the presence of non-viral nucleotides at the 3′ ends of viral transcripts may also reduce their biological activity. This effect is, in general, dependent on the length of the non-viral extension, with short extensions having little effect on infectivity (Boyer and Haenni, 1994). In an attempt to avoid the negative effects on infectivity caused by extra non-viral nucleotides at the 3′ ends of *in vivo* transcripts, the sequences of a number of ribozymes, including hepatitis delta virus (HDV) ribozyme, were positioned behind the viral sequences in the infectious clones to mediate their self-cleavage and produce (quasi) proper viral ends. In some cases where this has been analyzed, a positive influence on infectivity was reported when a ribozyme sequence was introduced in infectious clones. Turpen et al. (1993) found a two-fold increase in transfection frequency when a ribozyme based on subterranean clover mottle virus satellite RNA was included in an infectious tobacco mosaic virus clone. Similarly, Scholthof (1999) found the highest levels of infectivity with an infectious tomato bushy stunt virus clone when an HDV ribozyme was used in addition to the NOS terminator signal.

Although highly variable between experiments, typical infection rates of *N. benthamiana* plants with agroinfectious ToCV (Spanish isolate AT80/99-IC) clones rarely exceed 50% and despite our repeated attempts no tomato plants could be agroinfected (Orílio et al., 2014; our unpublished results). In this context, the objectives of this work were (i) to generate infectious cDNA clones for ToCV RNA1 and RNA2 containing a ribozyme at the 3′ end, (ii) to identify the 3′ ends of the virus genomes generated after agroinoculation with clones with or without the ribozyme, (iii) to quantify the effect of the ribozyme on the infection rate of *N. benthamiana* plants, and (iv) to determine whether a possible increase in infectivity due to the presence of the ribozyme would allow direct agroinfection of tomato plants.

## Materials and Methods

### Virus Isolate

Tomato chlorosis virus isolate AT80/99 was obtained from a naturally infected tomato plant collected in Málaga province, southern Spain, in 1999 (Lozano et al., 2006, 2007). Infectious ToCV clones (isolate AT80/99-IC, GenBank acc. nos. KJ740256 and KJ740257) have previously been developed under the control of the SP6 RNA polymerase promoter (pToCV227 [RNA1] and pToCV230 [RNA2]) and the 2× CaMV 35S promoter (pToCV-35S-RNA1 and pToCV-35S-RNA2) (Orílio et al., 2014).

### Construction of Infectious ToCV Clones Incorporating a Ribozyme Sequence

Clones pToCV227 and pToCV230 were used as templates to obtain the full-length sequences of ToCV RNA1 and RNA2 by PCR amplification, respectively. PCR was performed using PfuTurbo DNA polymerase, a mix of cloned Pfu DNA polymerase and ArchaeMaxx polymerase-enhancing factor (Agilent Technologies), and 5′ phosphorylated primers MA682/MA683 and MA684/MA685 (Table 1) based on AT80/99-IC RNA1 and RNA2 sequences, respectively. PCR products of the expected sizes were purified with a Zymoclean Gel DNA Recovery Kit (Zymo Research) and cloned into *Sma*I- and *Stu*I-digested pJL89, a binary vector from the pJL series constructed by Lindbo (2007), between the 2× CaMV 35S promoter and HDV ribozyme plus CaMV 35S terminator (Figure 1). Ligation products were transformed in *E. coli* strain ElectroMAX Stbl4 by electroporation (25 μF, 200 Ω, 1,200 V) in a Gene Pulser XCell (Bio-Rad). The presence of the correct inserts in clones selected by restriction analysis was confirmed by sequencing (Macrogen Inc., Seoul, South Korea) using primers based on the 2× CaMV 35S promoter (MA2720) and CaMV 35S terminator (MA2721) (Table 1). Clones pToCV-35S-RNA1-Rbz and pToCV-35S-RNA2-Rbz were selected for transformation into *Agrobacterium tumefaciens* strain GV3101 by electroporation as described above.

**TABLE 1:** List of primers used in this study.

Primer	Sequence (5′-3′)	Position in the ToCV genome^a^	Use
MA682	**^P^**GAAATAGTATTCGTGTGATTACAC	1–24	RNA1 amplification
MA683	**^P^**CGACCTATTTATTTATATACTAGATC	8,594–8,569	RNA1 amplification
MA684	**^P^**GAAATACAAGTCCAGGTGTTTCCTGTG	1–27	RNA2 amplification
MA685	**^P^**GACCTATTTATTTATATACTAAATCTACC	8,241–8,213	RNA2 amplification
MA2720	TAAGGGATGACGCACAATCC	–	RNA1/RNA2 5′ end sequencing (CaMV 35S promoter)
MA2721	CCCTTATCTGGGAACTACTC	–	RNA1/RNA2 3′ end sequencing (CaMV 35S terminator)
MA324	GRACDGGDTCDCCRAAYACKTGG	7,120–7,142	RNA1 3′ end cloning
MA2813	GGCTTGTCAAGACATTCTTGAGGG	8,012–8,035	RNA1 3′ end cloning
MA2798	GGAGTGTTTCACAAGATACGGG	7,484–7,505	RNA2 3′ end cloning
MA2799	GTTGCTCCATTCGGGAGTAACAGG	7,976–7,999	RNA2 3′ end cloning
MA279	CCGGATCCTCTAGAGCGGCCGCT_17_V	–	RNA1/RNA2 3′ end cloning
Oligo(dT)-anchor	GACCACGCGTATCGATGTCGACT_16_V	–	RNA1/RNA2 3′ end cloning
Anchor	GACCACGCGTATCGATGTCGAC	–	RNA1/RNA2 3′ end cloning

*^a^Nucleotide positions refer to the sequences of ToCV isolate AT80/99-IC (GenBank acc. nos. KJ740256 for RNA1 and KJ740257 for RNA2).*

### Plant Agroinoculation

For agroinoculation experiments, *A. tumefaciens* GV3101 carrying the constructs pToCV-35S-RNA1-Rbz or pToCV-35S-RNA2-Rbz were grown in Luria-Bertani broth containing rifampicin (25 μg/mL), gentamicin (20 μg/mL), tetracycline (2.5 μg/mL), kanamycin (50 μg/mL), 10 mM of 4-morpholineethanesulfonic acid (MES), and 20 mM of acetosyringone with vigorous shaking at 28°C for 48 h. Bacterial cells were harvested by centrifugation and resuspended in induction buffer (10 mM of MgCl_2_, 10 mM of MES pH 5.6, 10 mM of MgCl_2_, 150 mM of acetosyringone) to a final OD_600_ of 1.0. Both bacterial suspensions were mixed in a 1:1 ratio and then kept at room temperature for 2 h without shaking. In some experiments the OD_600_ = 1.0 cultures were diluted 1/50 and incubated for 2 h prior to inoculation. *A. tumefaciens* cultures carrying the constructs pToCV-35S-RNA1 and pToCV-35S-RNA2 (Orílio et al., 2014) or empty vectors (pCAMBIA2300 or pJL89) were used as positive and negative (mock) controls, respectively. *N. benthamiana* plants were agroinfiltrated at the 3–5 leaf stage on the underside of leaves with a 1 mL syringe without a needle. Tomato plants (cv. Moneymaker and cv. Micro-Tom) were inoculated by agroinfiltration at the 2–3 leaf stage as described for *N. benthamiana* or by agroinjection with a 27-gauge needle in the apex of the seedlings. Agroinoculated plants were maintained in a controlled temperature, insect-free growth chamber (25°C day/18°C night, 70% relative humidity, 16 h photoperiod at 250 μmol s^–1^ m^–2^ photosynthetically active radiation).

### Detection of Viral RNA in Agroinoculated Plants

The presence of ToCV RNA in non-inoculated leaves of agroinoculated plants was determined by tissue blot molecular hybridization 30 days post-inoculation (dpi). Freshly cross-sectioned leaf petioles were squash-blotted on positively charged nylon membranes (Roche Diagnostics) and hybridized with a ToCV-specific digoxigenin-labeled negative-sense RNA probe obtained from the coat protein gene (Fortes et al., 2012). The cross-linked RNA was hybridized at 65°C overnight in standard hybridization buffer [5×SSC (750 mM of NaCl, 75 mM of sodium citrate, pH 7.0), 0.1% N-lauryl sarcosine, 0.02% sodium dodecyl sulfate, and 2% blocking reagent (Roche Diagnostics)] with 50% formamide, followed by high stringency washing (65°C in 0.1× SSC, 0.1% sodium dodecyl sulfate) following standard procedures. Chemiluminescent hybridization signals were detected on X-ray film (Mamoray HT, Agfa) after treatment with CDP-Star (Roche Diagnostics).

### Cloning and Sequencing of the 3′ Ends of ToCV RNA1 and RNA2 in Agroinoculated *N. benthamiana* Plants

Total RNA was extracted from infected leaves of six (three agroinoculated with pToCV-35S-RNA1 + pToCV-35S-RNA2 and three with pToCV-35S-RNA1-Rbz + pToCV-35S-RNA2-Rbz) *N. benthamiana* plants 30 dpi using TRIsure Reagent (Bioline) as described by the manufacturer. The sequences of primers used in this study, including those based on the sequence of ToCV isolate AT80/99-IC, are listed in Table 1. To generate cDNA of the RNA1 3′ end, total RNA was polyadenylated using yeast USB poly (A) polymerase (USB Corporation). Reverse transcription was performed with SuperScript III reverse transcriptase (Invitrogen) and oligo(dT) primer MA297 using the positive-strand RNA as the template. Then, PCR amplification was conducted with Biotaq DNA polymerase (Bioline) using the nested primers MA324/MA279 and MA2813/MA279. cDNA of the RNA2 3′end was obtained using the 5′/3′ RACE Kit 2nd Generation (Roche Diagnostics) and oligo(dT)-anchor primer using the negative-strand RNA as the template. The RNA2 cDNA was purified with the High Pure PCR Product Purification Kit (Roche Diagnostics) and the d(A)-tail was added using Recombinant Terminal Transferase. Finally, PCR amplification was conducted with the Expand High Fidelity PCR System (Roche Diagnostics) using the nested primers MA2798/Anchor and MA2799/Anchor. DNA bands with the expected size were extracted from agarose gels, purified using the QIAquick Gel Extraction Kit (QIAGEN), cloned in the pGEM-T Easy System (Promega), and transformed into *E. coli* DH5α by electroporation (25 μF, 200 Ω, 1,200 V) with a Gene Pulser XCell electroporation system (Bio-Rad). Three clones were selected for each plant and sequenced at Macrogen Inc. (Seoul, South Korea) using T7 and SP6 promoter primers. DNA sequences were analyzed using the SeqMan software included in the DNASTAR package (Lasergene).

## Results and Discussion

### Construction of ToCV RNA1 and RNA2 cDNA Clones Incorporating an HDV Ribozyme

With the objective of improving the infectivity of available ToCV agroinfectious clones (Orílio et al., 2014), an HDV ribozyme sequence was incorporated at the end of both RNA1 and RNA2 by cloning in the binary vector pJL89 (Lindbo, 2007). This vector is designed to accommodate viral cDNA inserts able to produce *in vivo*-transcribed genome copies without, theoretically, extra non-viral nucleotides, an important point when attempting to obtain highly infectious transcripts (Boyer and Haenni, 1994). pJL89 vector has been successfully used to generate infectious clones of RNA plant viruses including two important pathogens of tomato: tomato marchitez virus (genus *Torradovirus*) (Ferriol et al., 2016) and pepino mosaic virus (genus *Potexvirus*) (Ruiz-Ramón et al., 2019).

In this work, full-length ToCV RNA1 and RNA2 sequences were obtained by PCR amplification using primers corresponding to the exact 5′ and 3′ ends of the viral sequence using high-fidelity DNA polymerases. To avoid the presence of extra 5′ non-viral nucleotides, the duplicated CaMV 35S promoter was positioned immediately upstream of the first nucleotide of the viral sequences, both for RNA1 and RNA2. On the other hand, the HDV ribozyme sequence, followed by the CaMV terminator sequence, was positioned immediately downstream of the viral cDNA with the objective of generating authentic viral 3′ ends. In an attempt to avoid the instability and toxicity problems observed with infectious closterovirid cDNA clones (Satyanarayana et al., 2003), ligation products were transformed into *E. coli* strain Stbl4, specifically designed for cloning unstable inserts. Recombinant plasmids pToCV-35S-RNA1-Rbz and pToCV-35S-RNA2-Rbz (Figure 1) were selected after confirming the size and sequence of the insert ends, and transformed into *A. tumefaciens* to be tested in plants.

### Improved Infectivity of Agroinfectious ToCV Clones in *N. benthamiana*

**FIGURE 2:**
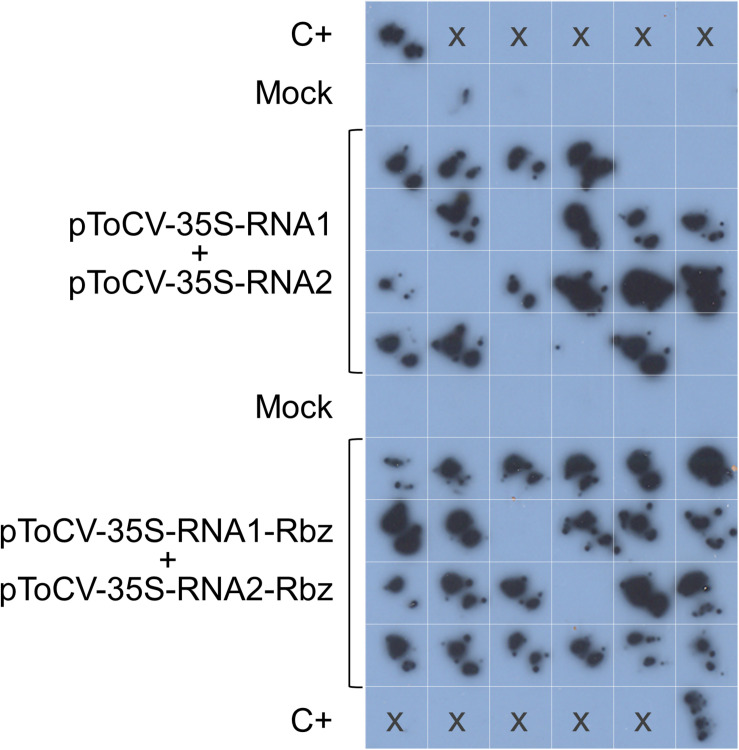
Agroinoculation experiments with tomato chlorosis virus. *Nicotiana benthamiana* plants were inoculated with *Agrobacterium tumefaciens* GV3101 cultures carrying pToCV-35S-RNA1 + pToCV-35S-RNA2 or pToCV-35S-RNA1-Rbz + pToCV-35S-RNA2-Rbz (Experiment 2 in Table 2). A specific digoxigenin-labeled RNA probe was used to analyze the agroinoculated *N. benthamiana* plants by molecular hybridization after tissue printing on nylon membranes. Tissue prints of mock-inoculated plants were included as negative controls. C+, ToCV-infected *N. benthamiana* plant used as positive control; x, no sample.

The biological activity of the new ToCV clones containing the HDV ribozyme downstream of the viral genome components (pToCV-35S-RNA1-Rbz + pToCV-35S-RNA2-Rbz) was first compared with that of previously available clones lacking the ribozyme sequence (pToCV-35S-RNA1 + pToCV-35S-RNA2) (Orílio et al., 2014), by agroinoculation of *N. benthamiana* plants. For this, systemic infection was assessed by tissue printing hybridization of non-infiltrated apical leaves using a negative sense CP gene probe. Four independent experiments were carried out, each including 6–24 plants per condition. Figure 2 shows the results of the molecular hybridization conducted in Experiment 2. In all experiments, the infectivity of the clones carrying the ribozyme, measured as the number of infected plants, was higher than that of the previously available clones. Considerable variability was observed between experiments, with the increase in infectivity ranging from 130 to 300% (Table 2). Considering all the experiments together, 82.1% of the plants inoculated with the clones containing the ribozyme became infected, whereas only 40.9% of the plants became infected with the previously available clones, thus resulting in a two-fold increase in infectivity. Similar heterogeneity of results between experiments was reported for infectious clones of the criniviruses LIYV (Wang et al., 2009) and LCV (Chen et al., 2012). A few studies of plant viruses belonging to different families also showed an increase in infectivity, measured as number of local lesions, when a ribozyme was incorporated into infectious clones. Examples include tobacco mosaic virus plus subterranean clover mottle virus satellite RNA ribozyme (Turpen et al., 1993) and tomato bushy stunt virus plus HDV ribozyme (Scholthof, 1999).

**TABLE 2:** Agroinoculation experiments carried out with infectious tomato chlorosis virus RNA1 and RNA2 clones (isolate AT80/99-IC) with or without the HDV ribozyme (Rbz).

		No. infected plants/no. inoculated plants (% infected plants)			
Host	Experiment	pToCV-35S-RNA1 + pToCV-35S-RNA2	pToCV-35S-RNA1-Rbz + pToCV-35S-RNA2-Rbz	Mock (empty vectors)	Notes
*N. benthamiana*	1	5/12 (41.66)	23/24 (95.83)	0/12 (0)	
	2	16/24 (66.66)	22/24 (91.66)	0/12 (0)	
	3	5/24 (20.83)	15/24 (62.50)	0/12 (0)	
	4	1/6 (16.66)	4/6 (66.66)	0/6 (0)	
Tomato	1	0/12 (0)	0/24 (0)	0/12 (0)	
	2	0/12 (0)	0/24 (0)	0/12 (0)	
	3	0/6 (0)	0/18 (0)	0/6 (0)	
	4	0/10 (0)	0/10 (0)	0/6 (0)	AI^a^
		0/10 (0)	0/10 (0)	0/6 (0)	AI, MT^b^
	5	0/10 (0)	0/10 (0)	0/6 (0)	AI, 1:50^c^
		0/10 (0)	0/10 (0)	0/6 (0)	AI, MT, 1:50

*Systemic infection was determined on agroinoculated and mock-inoculated Nicotiana benthamiana and tomato plants by tissue printing hybridization using a negative sense CP gene probe 30 days post-inoculation. Agroinfiltration, tomato cv. Moneymaker and A. tumefaciens OD = 1 were used unless otherwise specified. ^a^AI, agroinjection; ^b^MT, cv. Micro-Tom; ^c^1:50, A. tumefaciens OD = 1 diluted 1:50.*

### ToCV Clones Containing the HDV Ribozyme Are Still Not Agroinfectious in Tomato

Tomato chlorosis virus clones harboring the HDV ribozyme, which were shown to have increased infectivity in *N. benthamiana* plants, were further tested in tomato plants (Table 2). A total of 106 tomato plants were agroinoculated with pToCV-35S-RNA1-Rbz + pToCV-35S-RNA2-Rbz clones in five independent experiments. The experiments also included 70 tomato plants agroinoculated with the clones lacking the ribozyme (ToCV-35S-RNA1 + pToCV-35S-RNA2), previously reported to be agroinfectious in *N. benthamiana* but not in tomato (Orílio et al., 2014). The agroinoculation conditions assayed included leaf agroinfiltration and stem agroinjection, tomato cv. Moneymaker and cv. Micro-Tom, and different *A. tumefaciens* concentrations. None of the tomato plants agroinoculated with either clones became infected with ToCV according to tissue printing hybridization (data not shown).

Similarly to ToCV, most agroinfectious closterovirid clones are not able to directly infect the natural hosts but require prior infection of *N. benthamiana* as an intermediate host. Subsequently, the infectious virions can be transferred to the natural hosts to initiate an infection by bark-flap or stem slash inoculation (CTV; Gowda et al., 2005; Ambrós et al., 2011), whitefly transmission (LIYV; Wang et al., 2009), or grafting (ToCV; Orílio et al., 2014). It is worth mentioning that the cDNA clones generated for a Chinese ToCV isolate are also agroinfectious in *N. benthamiana* (Zhao et al., 2016) but not in tomato (Tao Zhou, personal communication).

Wang et al. (2009) suggested a number of factors that could contribute to the inability of agroinfectious viral clones to accomplish systemic infection in the natural host plants, including transcript splicing, thus reducing the functional RNA transcripts reaching the cytoplasm, the difference in virulence conferred by different *A. tumefaciens* strains, the plant defense responses caused by the combination of specific *A. tumefaciens* strains and viruses, the inaccessibility of phloem cells, or a strong silencing response at the very early stages of infection. Another possible factor is the difficulty coinfecting a single cell with several genome segments in the case of multipartite viruses such as criniviruses.

### Determination of 3′ End Viral Progeny Sequences

**FIGURE 3:**
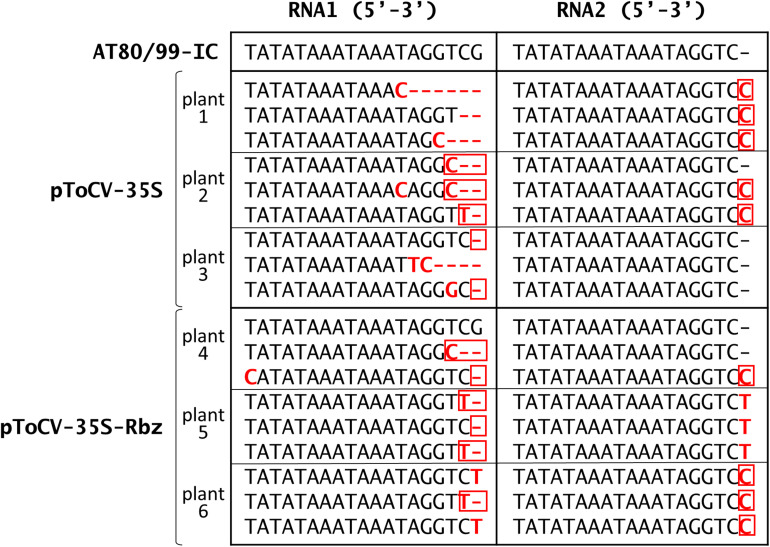
Sequencing of tomato chlorosis virus RNA1 and RNA2 3′ ends cloned from systemically infected *Nicotiana benthamiana* plants at 30 dpi. Plants were agroinoculated with pToCV-35S-RNA1 + pToCV-35S-RNA1 (pToCV-35S) or pToCV-35S-RNA1-Rbz + pToCV-35S-RNA2-Rbz (pToCV-35S-Rbz). Three plants were analyzed per condition and six clones (three for RNA1 and three for RNA2) were sequenced per plant. Nucleotide changes (deletions, insertions, or substitutions) with respect to the sequence of the infectious clones (isolate AT80/99-IC) are highlighted in bold and red. Of these, those nucleotide changes resulting in sequences present in other ToCV isolates (Table 3) are outlined in red.

In addition to analyzing the effect of positioning the HDV ribozyme behind the viral sequences in the infectious ToCV RNA1 and RNA2 clones on infectivity, which theoretically would be caused by the elimination of extra non-viral nucleotides at the 3′ ends of *in vivo* transcripts, the actual effect of the ribozyme on the viral progeny sequences was assessed. For this, the 3′ end viral progeny sequences present in the agroinoculated *N. benthamiana* plants were determined by cloning and sequencing at 30 dpi as a proxy for RNA sequences generated from the cDNA clones after HDV ribozyme self-cleavage. Three plants were analyzed per condition (pToCV-35S-RNA1 + pToCV-35S-RNA1 or pToCV-35S-RNA1-Rbz + pToCV-35S-RNA2-Rbz) and six clones (three for RNA1 and three for RNA2) were sequenced per plant (Figure 3). Variability was observed in the 3′ ends, mainly for RNA1, even among clones derived from the same plant. When compared with the sequence of the infectious clone (isolate AT80/99-IC), the very end of the RNA2 progeny showed 0–1 nucleotide differences regardless of whether clones with or without ribozyme were used. In contrast, differences were observed for RNA1 between the viral progeny present in plants agroinoculated with the clones with (0–3 nucleotide changes) or without (1–7 nucleotide changes) the ribozyme. Interestingly, most of the changes observed in the RNA2 progeny of the clones with the ribozyme resulted in sequences present in other ToCV isolates (Table 3), which could be considered as alternative proper viral ends. In summary, the presence of the HDV ribozyme produced RNA1 3′ ends more similar to those present in the clones used for agroinoculation or in other naturally occurring ToCV isolates. Sequence heterogeneity in the 3′ end of sequences of closterovirid isolates is not surprising, as this has been shown in CTV isolates, for example (López et al., 1998; Chen et al., 2018).

**TABLE 3:** Tomato chlorosis virus isolates available in GenBank with 3′ end nucleotide sequences identical to some of the variants detected in the viral progeny of agroinoculated *Nicotiana benthamiana* plants (Figure 3).

	RNA1		RNA2		
Isolate	Acc. no.	Sequence (5′-3′)	Acc. no.	Sequence (5′-3′)	References
AT80/99-IC	KJ740256	TAAATAAATAGGTCG	KJ740257	TAAATAAATAGGTC-	Orílio et al., 2014
ToC-Br2	JQ952600	TAAATAAATAGG**C–**	–	–	Albuquerque et al., 2013
2.5	KJ200304	TAAATAAATAGGT**T-**	–	–	Unpublished
MM8	KJ200306	TAAATAAATAGGTC**-**	–	–	Unpublished
Pl-1-2	KJ200308	TAAATAAATAGGTC**-**	–	–	Unpublished
XS	–	–	KY618797	TAAATAAATAGGTC**C**	Kang et al., 2018
FERA_160205	KY810786	TAAATAAATAGGTC**-**	–	–	Pecman et al., 2017

*Nucleotide changes present in the mentioned variants with respect to the sequences of the infectious clones (isolate AT80/99-IC, included for comparison) are highlighted in bold and underlined.*

## Conclusion

To our knowledge, this is the first time that the effect of the presence/absence of a ribozyme positioned immediately downstream from the viral genome in a cDNA clone on infectivity and 3′ end viral progeny sequences has been analyzed for a closterovirid. From a practical perspective, the novel infectious ToCV cDNA clones represent an advance as infectivity on *N. benthamiana*, an almost universal plant virus host that is very useful for studying basic aspects of the plant–virus interaction, increased by 100%. Also, the availability of these clones could form the basis for further approaches to achieve an important goal, the direct agroinfection of tomato plants, the primary host of ToCV, which would allow reverse genetic studies, understanding of the virus–vector interactions and facilitation of breeding programs to incorporate resistance against ToCV in commercial cultivars.

## Data Availability Statement

The original contributions presented in the study are included in the article, further inquiries can be directed to the corresponding authors.

## Author Contributions

JN-C and EF-O contributed to the conception and design of the study. EN-H performed the experiments and wrote the first draft of the manuscript, which was revised by JN-C and EF-O. All authors approved the final version of the manuscript.

## Conflict of Interest

The authors declare that the research was conducted in the absence of any commercial or financial relationships that could be construed as a potential conflict of interest.

## Publisher’s Note

All claims expressed in this article are solely those of the authors and do not necessarily represent those of their affiliated organizations, or those of the publisher, the editors and the reviewers. Any product that may be evaluated in this article, or claim that may be made by its manufacturer, is not guaranteed or endorsed by the publisher.

## References

[B1] Agranovsky A. A. (2016). “Closteroviruses: molecular biology, evolution and interactions with cells,” in *Plant Viruses: Evolution and Management*, eds Gaur R. K. Petrov N. M. Patil B. Stoyanova M. I. (Singapore: Springer), 231–252. 10.1007/978-981-10-1406-2_14

[B2] Albuquerque L. C. Villanueva F. Resende R. O. Navas-Castillo J. Barbosa J. C. Inoue-Nagata A. K. (2013). Molecular characterization reveals Brazilian *Tomato chlorosis virus* to be closely related to a Greek isolate. *Trop. Plant Pathol.* 38 332–336. 10.1590/S1982-56762013005000016

[B3] Ambrós S. El-Mohtar C. Ruiz-Ruiz S. Peña L. Guerri J. Dawson W. O. (2011). Agroinoculation of *Citrus tristeza virus* causes systemic infection and symptoms in the presumed nonhost *Nicotiana benthamiana*. *Mol. Plant Microbe Interact.* 24 1119–1131. 10.1094/MPMI-05-11-0110 21899435

[B4] Ambrós S. Ruiz-Ruiz S. Peña L. Moreno P. (2013). A genetic system for *Citrus tristeza virus* using the non-natural host *Nicotiana benthamiana*: an update. *Front. Microbiol.* 4:165. 10.3389/fmicb.2013.00165 23847598 PMC3698417

[B5] Amer M. A. Ibrahim Y. E. Kheder A. A. Hamed A. H. Farrag A. A. Al-Saleh M. A. (2020). Confirmation incidence of Tomato chlorosis virus naturally infecting tomato crop in Egypt. *Int. J. Agric. Biol.* 23 963–969. 10.17957/IJAB/15.1375 29653435

[B6] Boyer J. C. Haenni A. L. (1994). Infectious transcripts and cDNA clones of RNA viruses. *Virology* 198 415–426. 10.1006/viro.1994.1053 8291226

[B7] Cañizares M. C. Navas-Castillo J. Moriones E. (2008). Multiple suppressors of RNA silencing encoded by both genomic RNAs of the *Crinivirus*, *Tomato chlorosis virus*. *Virology* 379 168–174. 10.1016/j.virol.2008.06.020 18644612

[B8] Chen A. Y. S. Pavitrin A. Ng J. C. K. (2012). Agroinoculation of the cloned infectious cDNAs of *Lettuce chlorosis virus* results in systemic plant infection and production of whitefly transmissible virions. *Virus Res.* 169 310–315. 10.1016/j.virusres.2012.08.010 22926259

[B9] Chen A. Y. S. Watanabe S. Yokomi R. Ng J. C. K. (2018). Nucleotide heterogeneity at the terminal ends of the genomes of two California *Citrus tristeza virus* strains and their complete genome sequence analysis. *Virol. J.* 15:141. 10.1186/s12985-018-1041-4 30219073 PMC6139129

[B10] Ferriol I. Turina M. Zamora-Macorra E. J. Falk B. W. (2016). RNA1-independent replication and GFP expression from Tomato marchitez virus isolate M cloned cDNA. *Phytopathology* 106 500–509. 10.1094/PHYTO-10-15-0267-R 26756828

[B11] Fiallo-Olivé E. Navas-Castillo J. (2019). Tomato chlorosis virus, an emergent plant virus still expanding its geographical and host ranges. *Mol. Plant Pathol.* 20 1307–1320. 10.1111/mpp.12847 31267719 PMC6715620

[B12] Fortes I. M. Moriones E. Navas-Castillo J. (2012). Tomato chlorosis virus in pepper: prevalence in commercial crops in south-eastern Spain and symptomatology under experimental conditions. *Plant Pathol.* 6 994–1001. 10.1111/j.1365-3059.2011.02584.x

[B13] Fuchs M. Bar-Joseph M. Candresse T. Maree H. J. Martelli G. P. Melzer M. J. (2020). ICTV virus taxonomy profile: *Closteroviridae*. *J. Gen. Virol.* 101 364–365. 10.1099/jgv.0.001397 32134375 PMC7414439

[B14] Gowda S. Satyanarayana T. Robertson C. J. Garnsey S. M. Dawson W. O. (2005). “Infection of citrus plants with virions generated in *Nicotiana benthamiana* plants agroinfiltrated with a binary vector based *Citrus tristeza virus*,” in *Proceedings of the 16^*th*^ Conference of the International Organization of Citrus Virologists*, eds Hilf M. E. Duran-Vila N. Rocha-Peña M. A. (Riverside, CA: IOCV), 23–33.

[B15] Jarugula S. Gowda S. Dawson W. O. Naidu R. A. (2018). Development of infectious cDNA clones of *Grapevine leafroll-associated virus 3* and analyses of the 5′ non-translated region for replication and virion formation. *Virology* 523 89–99. 10.1016/j.virol.2018.07.023 30103103

[B16] Kang Y. C. Wang Y. C. Hsia C. M. Tsai W. S. Huang L. H. Yeh S. D. (2018). Molecular characterization and detection of a genetically distinct tomato chlorosis virus strain in Taiwan. *Plant Dis.* 102 600–607. 10.1094/PDIS-05-17-0728-RE 30673497

[B17] Kimathi R. H. Wilisiani F. Mashiko T. Neriya Y. Miinda A. E. Nishigawa H. (2020). First report of *Tomato chlorosis virus* infecting tomato in Kenya. *Sci. Afr.* 7:e00286. 10.1016/j.sciaf.2020.e00286

[B18] Kiss Z. A. Medina V. Falk B. (2013). *Crinivirus* replication and host interactions. *Front. Microbiol.* 4:99. 10.3389/fmicb.2013.00099 23730299 PMC3657685

[B19] Kurth E. G. Peremyslov V. V. Prokhnevsky A. I. Kasschau K. D. Miller M. Carrington J. C. (2012). Virus-derived gene expression and RNA interference vector for grapevine. *J. Virol.* 86 6002–6009. 10.1128/JVI.00436-12 22438553 PMC3372183

[B20] Lindbo J. A. (2007). TRBO: a high-efficiency tobacco mosaic virus RNA-based overexpression vector. *Plant Physiol.* 145 1232–1240. 10.1104/pp.107.106377 17720752 PMC2151719

[B21] Liu Y. P. Peremyslov V. V. Medina V. Dolja V. V. (2009). Tandem leader proteases of *Grapevine leafroll-associated virus 2*: host-specific functions in the infection cycle. *Virology* 383 291–299. 10.1016/j.virol.2008.09.035 19007962 PMC7103369

[B22] Livieratos I. C. Eliasco E. Müller G. Olsthoorn R. C. L. Salazar L. F. Pleij C. W. A. (2004). Analysis of the RNA of Potato yellow vein virus: evidence for a tripartite genome and conserved 3′-terminal structures among members of the genus *Crinivirus*. *J. Gen. Virol.* 85 2065–2075. 10.1099/vir.0.79910-0 15218192

[B23] López C. Ayllón M. A. Navas-Castillo J. Guerri J. Moreno P. Flores R. (1998). Molecular variability of the 5′- and 3′-terminal regions of citrus tristeza virus RNA. *Phytopathology* 88 685–691. 10.1094/PHYTO.1998.88.7.685 18944941

[B24] Lozano G. Moriones E. Navas-Castillo J. (2006). Complete nucleotide sequence of the RNA2 of the *Crinivirus* tomato chlorosis virus. *Arch. Virol.* 151 581–587. 10.1007/s00705-005-0690-y 16374719

[B25] Lozano G. Moriones E. Navas-Castillo J. (2007). Complete nucleotide sequence of the RNA1 of a European isolate of tomato chlorosis virus. *Arch. Virol.* 152 839–841. 10.1007/s00705-006-0886-9 17164961

[B26] Navas-Castillo J. Camero R. Bueno M. Moriones E. (2000). Severe yellowing outbreaks in tomato in Spain associated with infections of *Tomato chlorosis virus*. *Plant Dis.* 84 835–837. 10.1094/PDIS.2000.84.8.835 30832134

[B27] Orílio A. F. Fortes I. M. Navas-Castillo J. (2014). Infectious cDNA clones of the crinivirus *Tomato chlorosis virus* are competent for systemic plant infection and whitefly-transmission. *Virology* 46 365–374. 10.1016/j.virol.2014.07.032 25113907

[B28] Pecman A. Kutnjak D. Gutiérrez-Aguirre I. Adams I. Fox A. Boonham N. (2017). Next generation sequencing for detection and discovery of plant viruses and viroids: comparison of two approaches. *Front. Microbiol.* 8:1998. 10.3389/fmicb.2017.01998 29081770 PMC5645528

[B29] Prokhnevsky A. I. Peremyslov V. V. Napuli A. J. Dolja V. V. (2002). Interaction between long-distance transport factor and Hsp70-related movement protein of Beet yellows virus. *J. Virol.* 76 11003–11011. 10.1128/JVI.76.21.11003-11011.2002 12368343 PMC136651

[B30] Raza A. Shakeel M. T. Umar U. U. D. Tahir M. N. Hassan A. A. Katis N. I. (2020). First report of tomato chlorosis virus infecting tomato in Pakistan. *Plant Dis.* 104:2036. 10.1094/PDIS-12-19-2732-PDN

[B31] Ruiz-Ramón F. Sempere R. N. Méndez-López E. Sánchez-Pina M. A. Aranda M. A. (2019). Second generation of pepino mosaic virus vectors: improved stability in tomato and a wide range of reporter genes. *Plant Methods* 15:58. 10.1186/s13007-019-0446-4 31149024 PMC6537163

[B32] Satyanarayana T. Gowda S. Ayllón M. A. Dawson W. O. (2003). Frameshift mutations in infectious cDNA clones of *Citrus tristeza virus*: a strategy to minimize the toxicity of viral sequences to *Escherichia coli*. *Virology* 313 481–491. 10.1016/S0042-6822(03)00387-8 12954215 PMC7125997

[B33] Scholthof H. B. (1999). Rapid delivery of foreign genes into plants by direct rub-inoculation with intact plasmid DNA of a tomato bushy stunt virus gene vector. *J. Virol.* 73 7823–7829. 10.1128/JVI.73.9.7823-7829.1999 10438874 PMC104311

[B34] Shi Y. Shi Y. Gu Q. Yan F. Sun X. Li H. (2016). Infectious clones of the crinivirus cucurbit chlorotic yellows virus are competent for plant systemic infection and vector transmission. *J. Gen. Virol.* 97 1458–1461. 10.1099/jgv.0.000453 26982585

[B35] Turpen T. H. Turpen A. M. Weinzettl N. Kumagai M. H. Dawson W. O. (1993). Transfection of whole plants from wounds inoculated with *Agrobacterium tumefaciens* containing cDNA of tobacco mosaic virus. *J. Virol. Methods* 42 227–240. 10.1016/0166-0934(93)90035-P 8514842

[B36] Tzanetakis I. E. Martin R. R. Wintermantel W. (2013). Epidemiology of criniviruses: an emerging problem in world agriculture. *Front. Microbiol.* 4:119. 10.3389/fmicb.2013.00119 23730300 PMC3656352

[B37] Wang J. Turina M. Stewart L. R. Lindbo J. A. Falk B. W. (2009). Agroinoculation of the *Crinivirus*. Lettuce infectious yellows virus, for systemic plant infection. *Virology* 392 131–136. 10.1016/j.virol.2009.06.034 19632699

[B38] Wintermantel W. M. Wisler G. C. Anchieta A. G. Liu H. Y. Karasev A. V. Tzanetakis I. E. (2005). The complete nucleotide sequence and genome organization of tomato chlorosis virus. *Arch. Virol.* 150 2287–2298. 10.1007/s00705-005-0571-4 16003497

[B39] Wisler G. C. Duffus J. E. Liu H. Li R. H. (1998a). Ecology and epidemiology of whitefly-transmitted closteroviruses. *Plant Dis.* 82 270–280. 10.1094/PDIS.1998.82.3.270 30856856

[B40] Wisler G. C. Li R. H. Liu H. Y. Lowry D. S. Duffus J. E. (1998b). Tomato chlorosis virus: a new whitefly-transmitted, phloem limited, bipartite closterovirus of tomato. *Phytopathology* 88 402–409. 10.1094/PHYTO.1998.88.5.402 18944918

[B41] Zhao R. Wang N. Liu S. Ling K. S. Fan Z. Zhou T. (2016). P22 of tomato chlorosis virus, an RNA silencing suppressor, is naturally expressed in the infected plant. *Acta Virol.* 60 423–425. 10.4149/av_2016_04_423 27928924

